# Development and psychometric evaluation of the assessment of self-injection questionnaire: an adaptation of the self-injection assessment questionnaire

**DOI:** 10.1186/s12955-020-01606-7

**Published:** 2020-11-04

**Authors:** Farrah Pompilus, Anna Ciesluk, Sara Strzok, Valerie Ciaravino, Kristina Harris, Boglarka Szegvari, Irina Mountian, Sophie Cleanthous, Juliette Meunier

**Affiliations:** 1 Modus Outcomes, 1 Broadway, Cambridge, MA USA; 2 UCB Pharma, Lyon, France; 3 UCB Pharma, Hong Kong, Hong Kong; 4 UCB Pharma, Brussels, Belgium; 5 Modus Outcomes, Letchworth Garden City, UK; 6 Modus Outcomes, Lyon, France

**Keywords:** Patient-reported outcomes, Self-injection, Psychometric analysis, Rheumatology, Exit interviews, Focus groups, Concept elicitation, Cognitive debriefing

## Abstract

**Background:**

Patient-reported outcome (PRO) instruments provide robust and effective means of evaluating patients’ treatment experience; however, none adequately cover experience using self-injection devices with enhanced features, such as an electromechanical autoinjector (e-Device). The aim of this study was to develop a PRO instrument that accurately assesses patient experience of using an e-Device and to evaluate its psychometric properties.

**Methods:**

A mixed-methods approach was taken; two parallel, targeted literature reviews were conducted to identify relevant concepts and existing self-injection PRO instruments that could be adapted. Patient feedback obtained from two focus groups was used to inform initial instrument development. The pilot instrument was then administered in a multicenter, open-label, phase 3 clinical study in which patients self-injected certolizumab pegol using an e-Device, to gather evidence of its psychometric qualities. Exit interviews were conducted with a sub-sample of patients enrolled in the study to confirm the appropriateness and clarity of the items included and cognitively debrief the instrument. Confirmatory factor analysis (CFA) was conducted on all items, and each domain’s internal consistency was measured using Cronbach’s ɑ.

**Results:**

The literature searches identified several e-Device-specific concepts related to device features, device function, side effects/reactions/pain, confidence, and interference/convenience in daily life. Seven existing PRO instruments were identified. The Self-Injection Assessment Questionnaire (SIAQ), containing pre- and post-injection questionnaire modules, was selected as most suitable and adapted using feedback from 19 patients in the two focus groups to form the pilot Assessment of Self-Injection (ASI) questionnaire. CFA resulted in some changes to the grouping of items in the post-injection module domains following psychometric evaluation of the ASI. Internal consistency was satisfactory for all pre- and post-injection domains (ɑ > 0.8). Cognitive debriefing results from 12 patient exit interviews confirmed the ASI’s appropriateness and clarity.

**Conclusions:**

The ASI was developed iteratively with patient input and was evaluated in its intended clinical context of use. Psychometric analyses indicated promising cross-sectional results; the ASI was well understood and considered relevant by patients self-injecting using the e-Device, suggesting that it could be used in real-world settings to aid with clinical decision making.

*Trial registration:* NCT03357471

## Introduction

Self-injection devices can directly impact patient treatment experience; by optimizing the device it may be possible to improve treatment adherence and resultant clinical outcomes [1]. Medical devices for self-injecting treatments are continually evolving to improve patient experience; new devices have been designed to incorporate functionalities that specifically target the needs of patients, such as injection reminders, and injection speed control [1–3]. Evidence supports that treatment experience may be improved by providing a portfolio of different injection devices, from which patients can select the device most suited to their individual needs [4–6].

Anti-tumor necrosis factor (anti-TNF) biologics are established and effective treatments for a number of chronic inflammatory diseases including rheumatoid arthritis (RA), axial spondyloarthritis (axSpA), psoriatic arthritis (PsA), psoriasis (PSO), and Crohn’s disease (CD). Most anti-TNF agents are administered subcutaneously, often by self-injection [7]. Despite acceptable treatment safety and efficacy profiles, treatment adherence is often sub-optimal [3, 8]. Certolizumab pegol (CZP) is an Fc-free, PEGylated anti-TNF approved for the treatment of adult patients with moderate to severe RA, axSpA, PsA, PSO and CD [9, 10]. In the European Union, CZP is administered by subcutaneous self‑injection via pre-filled syringe (PFS), pre-filled pen (PFP), and ava^®^, a newly‑approved electromechanical auto-injection device (e-Device) [1, 9, 11].

Patient-reported outcome (PRO) instruments provide a means of assessing patient perception of their health status and treatment experience, and can be used to assess experience using new devices. Although existing PRO instruments specifically designed for self-injection devices are available, none adequately capture the patient experience of using more recently developed devices with advanced features. Questions relating to patient experience, ease-of-use, and safety need to address all aspects of injecting, including usefulness of injection logging and reminders, technique, accuracy of injection speed, and needle disposal, to ascertain specific feedback which can be used to aid clinical decision-making and to inform and improve device development. The aim of this study was to develop a PRO instrument, through adaptation of an existing instrument, that accurately assesses all aspects of patient experience of self-injection using an e-Device, such as ava^®^, and to generate evidence for its psychometric evaluation.

## Methods

### Overview

**Fig. 1:**
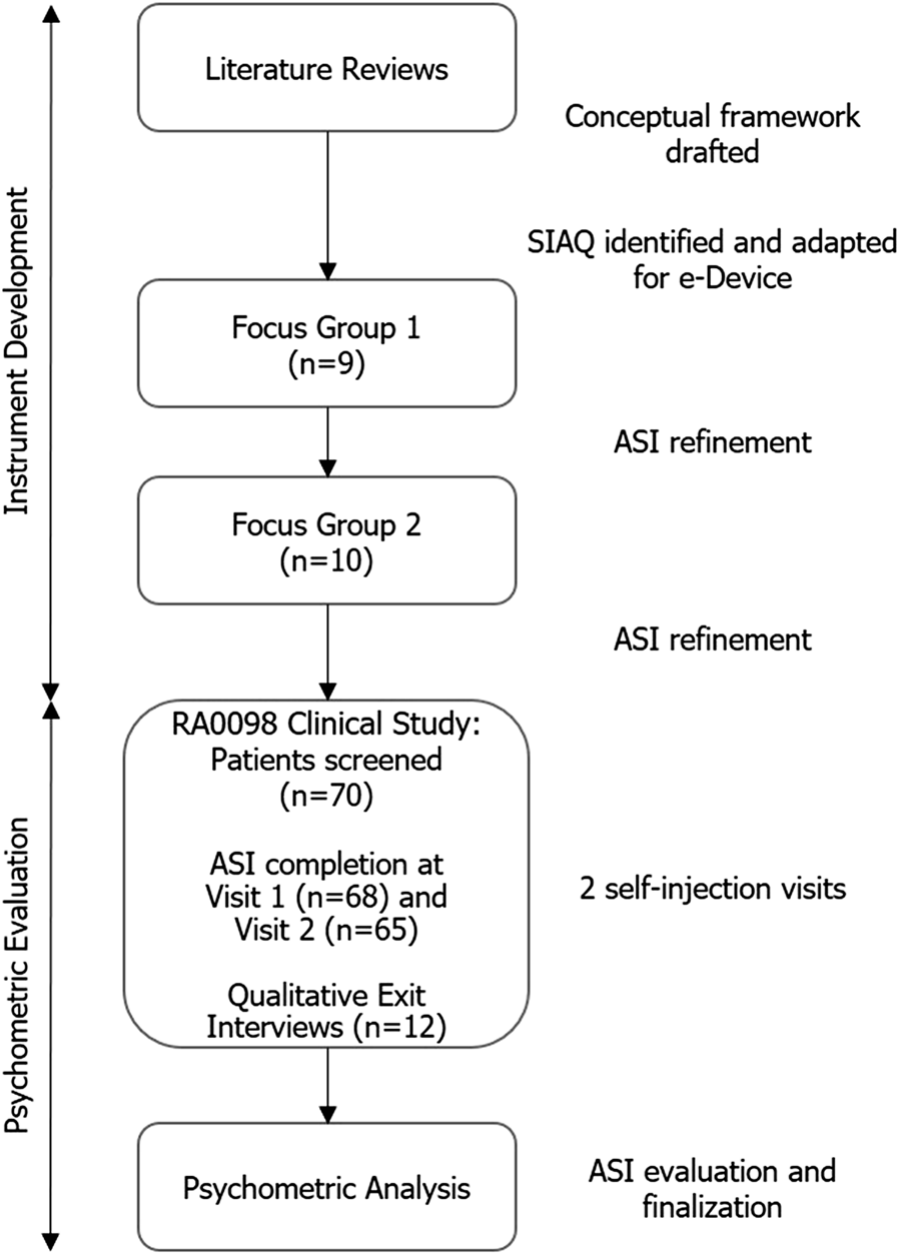
Overview of PRO development. *ASI* Assessment of Self-Injection, *PRO* patient-reported outcome, *SIAQ* Self-Injection Assessment Questionnaire

A mixed methods approach was taken (Fig. 1) [12]. This included targeted literature reviews to identify key concepts relating to self-injection devices and relevant existing PRO instruments; patient-centered qualitative research to confirm conceptualization and refine the pilot PRO; and psychometric analysis of clinical study PRO data to evaluate its psychometric properties. This research was carried out in alignment with the recommendations and best scientific practices described in the US Food and Drug Administration PRO Guidance [13, 14].

### Literature review and conceptual framework development

Two parallel, targeted literature reviews were conducted in PubMed in March 2015 to identify concepts relevant to patients who self-inject their treatment and existing PRO instruments used to assess patient experience using self-injection devices. The search terms and inclusion/exclusion criteria used are listed in Additional file 1: Table S1.

The concept literature review identified proximal and distal factors related to the benefits of injection devices. Proximal factors included those specific to using the device, whereas distal factors included potential practical and emotional factors relating to the use of device. Both clusters of concepts can impact device user satisfaction, acceptability and preference. The results of the concept literature review were inductively integrated into a conceptual framework, outlining key aspects of self-injection experience, including those likely to arise when using an e-Device. The conceptual framework was further supplemented with internal sponsor-conducted research on device attributes that could impact patient experience. Item-to-concept mapping compared item content of identified existing PRO instruments to the conceptual framework to assess each instrument’s conceptual coverage and content validity. Thus, item-to-concept mapping helped determine the most suitable PRO instrument(s) for adaptation to the e-Device context, as well as the gaps that would need to be addressed.

### Patient-centered research: PRO adaptation and refinement

Two sequential, semi-structured patient focus groups moderated by two trained interviewers were conducted to gain feedback on the new pilot instrument. Ethical approval was not required for these focus groups as the process was considered market research. For inclusion, patients were aged ≥ 18 years and had confirmed diagnosis of moderate to severe RA, axSpA, PsA or AS. Independent recruiters were instructed to include a mix of male/female patients, age groups, education levels, and both injection-experienced and injection-naïve patients who had a variety of relevant conditions. Questions were designed to explore self-injection concepts important to patients; initial open-ended questions were followed with probe questions to clarify feedback as necessary. To support the discussion, patients had the opportunity to examine both the PFS and e-Device, ask questions as to how they operate and their intended use, and watch a simulated “self-injection event” using the PFS on a fake skin pad. After concept elicitation, cognitive debriefing activities examined the clarity and relevance of all instructions, items and response options included in the pilot PRO instrument [15].

The pilot instrument was refined using the patient feedback and administered in RA0098 (NCT03357471), a multicenter, open-label, phase 3 clinical study conducted in the USA that assessed the ability of adult patients already self-injecting CZP using the PFS to safely and effectively self-inject CZP using ava^®^ [16]. Administering the instrument in RA0098 enabled qualitative review and evaluation of its psychometric properties. To support the instrument’s content validity, targeted concept elicitation and cognitive debriefing was undertaken with a sub-sample of patients enrolled in RA0098 who agreed to participate in exit interviews. In these interviews, patients answered open-ended questions about their experience of using ava^®^ and completed the pilot questionnaire online. Patients concurrently provided feedback to a trained interviewer over the phone regarding the instrument’s clarity, relevance and interpretability.

Transcribed interviews were coded and qualitatively analyzed by independent experts to determine whether all important concepts had been covered appropriately. Coded transcripts were ordered chronologically, based on interview completion dates, and then divided into five sequential groups. New concepts that emerged from the second group were compared with the concepts that emerged in the transcripts from the first group. This evaluation and comparison was repeated for each additional group. The cycle of data collection and analysis continued until further data collection produced minimal or no new relevant concepts related to the ava^®^ e-Device experience.

### Psychometric evaluation of the pilot PRO

#### Study design

As noted above, RA0098 assessed the safe and effective use of ava^®^ by patients (≥ 18 years) diagnosed with moderate to severe RA, PsA, AS, or CD and self-injecting CZP using the PFS. Patients received training and self-injected CZP using the e-Device at two visits, separated by either two weeks (Q2W; 1 × 200 mg CZP) or four weeks (Q4W; 2 × 200 mg CZP), in accordance with their previous dosing schedule. An International Review Board/Independent Ethics Committee approved the study protocol and an Informed Consent form, which was signed by all patients prior to the start of the study.

#### Construct validity

Confirmatory factor analysis (CFA) assessed the content structure of the PRO instrument at Visit 1 to confirm whether items adequately contributed to a given domain. This was conducted to align with the development process of an existing self-injection PRO instrument [17]. Any items with a standardized loading estimate < 0.6 were considered for removal from the domain since their relation to the measured domain would be considered weak. The goodness-of-fit of the CFA model was considered using the following: root mean square error of approximation (RMSEA), for which RMSEA < 0.05 was considered a good fit or acceptable fit if RMSEA < 0.08; root mean square residual (RMR) and standardized RMR, for which RMR < 0.05 was considered a good fit; goodness of fit index (GFI) and adjusted GFI (AGFI), for which GFI or AGFI > 0.90 was considered a good fit; normed fit index (NFI) and comparative fit index (CFI), for which NFI or CFI > 0.90 was considered a good fit. Cross-sectional Rasch measurement theory (RMT) analysis measured the extent to which the observed data (patients’ responses to scale items) “fit” the predictions for those responses generated from the Rasch model which defines how a set of items should perform to generate reliable and valid measurements. RMT analysis was performed on all PRO data pooled from Visit 1 and Visit 2.

#### PRO scoring

Hypothesized domains of the pilot PRO were the same of those of the existing self-injection PRO instrument, with the same items in each domain. Potential item reduction within each domain of the pilot PRO was based on the items’ acceptability and applicability (description of the items), redundancy (CFA, RMT analysis) and targeting (RMT analysis).

As there are 4 to 6 response options for the items, item scores were all transformed on a scale ranging from 0 to 10 before calculating domain scores, to ensure all items used the same scale to allow comparison. Domain scores were calculated as the mean of the recoded items scores if ≥ 50% of the items in the domain were completed. For the pre-injection module, for Feelings About Injection domain, higher scores indicated more negative feelings; for Self-Confidence, higher scores indicated more confidence; and for Satisfaction, higher scores indicated higher satisfaction. For the post-injection module; for Feeling about Injections, higher scores indicated feeling more afraid or anxious; for Self-Image, higher scores indicated feeling more self-conscious; for Self-Confidence, higher scores indicated higher confidence; for Pain and Skin Reactions, higher scores indicated more severe reactions; for Ease of Use, higher scores indicated easier use ratings; for Satisfaction, higher scores indicated greater satisfaction.

#### Psychometric analysis

Floor and ceiling effects (i.e. the proportion of respondents scoring the lowest or highest possible scores) were calculated for all scores at each visit. Internal consistency for each domain was evaluated using Cronbach’s alpha (ɑ) at Visit 1. Within its range of 0.0–1.0, an ɑ > 0.70 is the recommended minimum value for group comparisons (though a minimum value > 0.80 is desired) [18, 19]. Convergent and discriminant validity was assessed using Spearman correlation coefficients between items and scores; items should show a higher correlation with their own domain than with the other domains and their correlation with their domain should be at least moderate (≥ 0.40). Known-groups validity was assessed for age group, gender, disease, and dosing schedule group; no a priori hypotheses were defined as this analysis was exploratory. Test–retest reliability was assessed between visits; the difference between overall scores of the pilot instrument’s two modules at Visit 1 and Visit 2 were compared using paired t-tests. Intraclass correlation coefficients were calculated between scores at Visit 1 and scores at Visit 2. Intraclass correlation coefficients > 0.70 are recommended for the scores to be considered reliable [20]. Responsiveness (ability to detect change) was assessed by characterizing the change from Visit 1 to Visit 2 across all patient sub-groups using two effect size calculations (Kazis’ effect size [ES] and standardized response mean [SRM]) [21, 22].

All analyses were performed using SAS software version 9.4 (SAS Institute Inc., Cary, NC, USA), except the RMT analysis that was performed using RUMM version 2030 (RUMM Laboratory Pty Ltd., Perth, Australia).

## Results

### Literature review and development of conceptual framework

#### Identified concepts relevant to e-Device-specific features

**Fig. 2:**
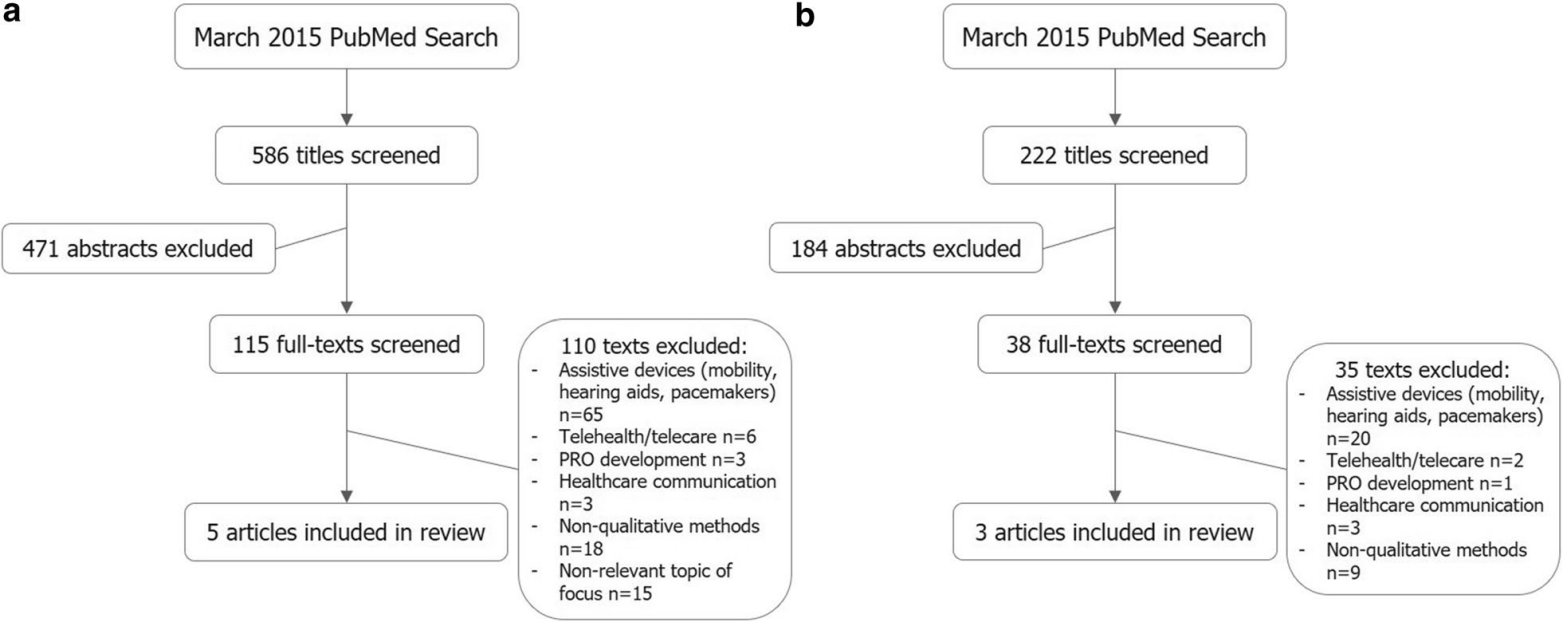
Literature review searches. **a** Clinical, observational study concepts literature review. **b** PRO instrument literature review. *PRO* patient-reported outcome

**Fig. 3:**
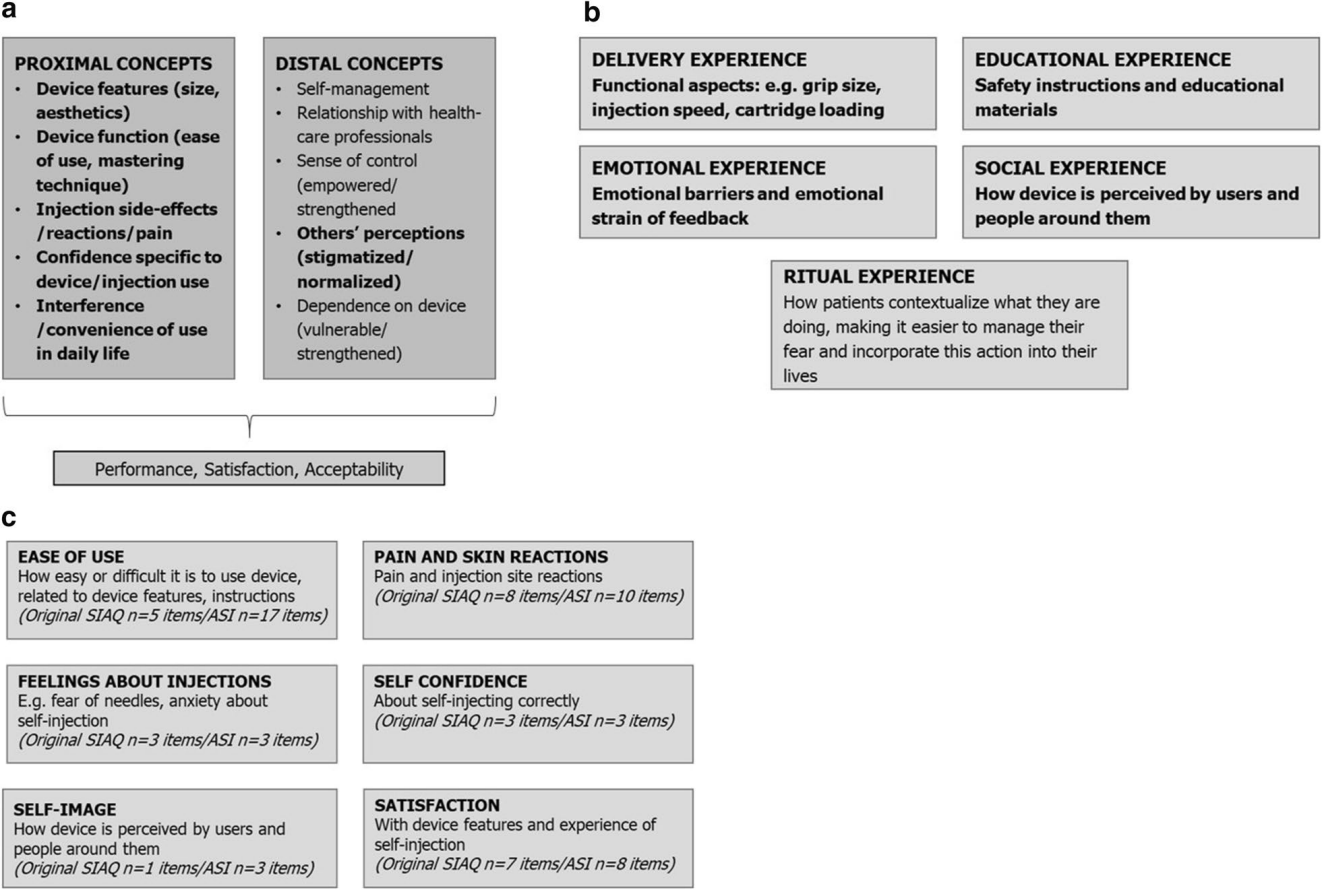
Self-injection conceptual framework development. **a** Literature-informed conceptual framework. **b** Sponsor-informed conceptual framework. Bold concepts incorporated into final ASI conceptual framework. **c** Final ASI conceptual framework, adapted from Keininger and Coteur, 2011. *ASI* Assessment of Self-Injection, *SIAQ* Self-Injection Assessment Questionnaire

Of the 586 clinical studies identified and screened, 5 included concepts relevant to patients who self-inject their medication; of the 222 observational studies identified and screened, 3 included concepts relevant to patients who self-inject their medication (Fig. 2). The literature review identified proximal concepts including device features, function, ease of use, mastering technique, side effects/reactions/pain, confidence, and interference/convenience in daily life; as well as distal concepts including self-management, sense of control and others’ perceptions of self-injection (Fig. 3). In parallel, a sponsor-informed conceptual framework was created based on expert internal consultation that included five main domains: delivery experience, educational experience, emotional experience, social experience, and ritual experience (Fig. 3).

#### Selection of PRO instrument for adaptation

Several relevant PRO instruments were identified: pre- and post-SIAQ [17]; IPAQ [23]; TRIM-Diabetes/Device [24]; TRIM-Diabetes MSTCQ [25]; and 6-item for RA Methotrexate [26]. Of these instruments, the Self-Injection Assessment Questionnaire (SIAQ) appeared to be the best candidate for adaptation to the e-Device context based on its conceptual coverage. The SIAQ comprises both pre- and post-injection modules, organized in a three- and six-domain conceptual framework (pre-injection: Feelings about Injections; Self-Confidence; and Satisfaction; followed by post-injection: Ease of Use; Pain and Skin Reactions; Feelings about Injections; Self-Confidence; Self-Image; and Satisfaction), which includes many of the important concepts identified from the literature review and sponsor research. However, the SIAQ did not cover features specific to an e-Device. For example, the SIAQ did not include aspects of patient-perceived benefit with using an e-Device, such as the ease of use of the display screen, cassette security, and injection speed control.

Based on concepts identified in the literature review and consideration of the properties of the e-Device identified by the sponsor, the conceptual framework was updated (Fig. 3). The SIAQ was then adapted to form an e-Device-relevant instrument, named the Assessment of Self-Injection (ASI; Additional file 3: Table S3). Several items were added to or deleted from the SIAQ to create the pilot version of the ASI (Additional file 4: Table S4). For example, items such as “bleeding at injection site” and “medication leaking at injection site” were added to the Pain and Skin Reactions domain, three items (“remove needle cap”; “hold at correct angle for injection”; “travel with device”) were added to the ease of use domain, and the “pause/stop” item also in this domain was split into two separate questions. Semantic changes were made to the Self-Confidence domain; feeling “embarrassed” or “uncomfortable” were replaced with “self-conscious” to remove ambiguity, and formatting changes were made throughout for improved readability and clarity. The pilot ASI was then tested and refined with input from patient focus groups.

### Patient-centered research: SIAQ adaptation and refinement

#### Focus groups

The first focus group was attended by nine patients with a variety of conditions (RA, axSpA, PsA, and CD) on September 11, 2015; mean age was 55.4 years [standard deviation (SD): 10.7 years], six were female and five had experience of self-injecting treatment (Table 1). Ten patients attended the second focus group on September 28, 2015, all with the same medical conditions; mean age was 44.9 years (SD: 9.6 years), seven were female and seven had experience of self-injecting treatment. Patients discussed advantages and disadvantages of an e-Device vs a PFS (Additional file 2: Table S2), and were asked to review and provide feedback on the pilot ASI. Most items (original and new) were well understood in both focus groups. Patients provided feedback and particularly endorsed the injection site reaction questions as diverse, comprehensive and relevant. The ASI was refined following each focus group; all patient feedback was incorporated, with two items merged and edited to form the item “how self-conscious would you feel if someone saw you with the (PFS/e-Device) (a) around family, (b) around your friends, (c) around people who you don’t know?.” The questionnaire was also reformatted (font, shading, spacing, etc.) to improve readability and ease of response (Additional file 4: Table S4).

**Table 1:** Focus group patient characteristics

Patient characteristics	Focus Group 1 (n = 9)	Focus Group 2 (n = 10)
*Diagnosis type, n (%)*		
RA	5 (55.6)	5 (50.0)
AS	2 (22.2)	2 (20.0)
PsA	1 (11.1)	3 (30.0)
axSpA	1 (11.1)	0 (0.0)
*Age, years*		
Mean (SD)	55.4 (10.7)	44.9 (9.6)
Minimum	40	32
Maximum	67	60
*Gender, n (%)*		
Female	6 (66.7)	7 (70)
Male	3 (33.3)	3 (30)
*Experience with self-injecting, n (%)*		
Yes	5 (55.6)	7 (70.0)
No	4 (44.4)	3 (30.0)

*AS* ankylosing spondylitis, *axSpA* axial spondyloarthritis, *PsA* psoriatic arthritis, *RA* rheumatoid arthritis, *SD* standard deviation

#### Exit interviews

A total of 12 patients with RA, PsA, or CD who completed the RA0098 clinical study participated in the exit telephone interviews. All interviews were conducted between January 3, 2018 and April 13, 2018 (Additional file 5: Table S5). Most patients were female (n = 10; 83%), and age ranged from 26 to 68 years, with a mean age of 51 years (SD: 13 years; Table 2). After codes were consolidated and merged, and codes not relevant to concepts of interest were removed, 44 spontaneously elicited concepts were identified for ava^®^ and its comparison to the PFS. Most concepts (n = 42; 95%) emerged within the first four groups; only two (5%) of these concepts emerged in the final saturation group. The two concepts were “inconvenience of having to store ava^®^ and its medication separately” and “no unwrapping needed for ava^®^ to access the medication.” Little information was obtained from the final interviews, which strongly suggests that conceptual saturation was achieved; concept elicitation results indicated that concepts important to patients self-injecting with an e-Device were well addressed in ASI. Cognitive debriefing confirmed that all items included in the pre- and post-injection modules of the ASI were appropriate and well understood, with patients reporting the following components as the most important (patients could endorse more than one item per questionnaire as “most important”): “anxiety of self-injecting” (n = 6); “confidence in self-injecting correctly” (n = 3), and “confidence in self-injecting safely” (n = 3) in the pre-injection module; “e-Device ergonomics/ease of use” (n = 5); “injection speed” (n = 3), and “overall convenience” in the post-injection module.

**Table 2:** Exit interview patient characteristics

*Diagnosis type, n (%)*	
RA	6 (50.0)
PsA	2 (16.7)
AS	2 (16.7)
CD	2 (16.7)
*Age, years*	
Mean (SD)	51 (13)
Minimum	26
Maximum	68
*Gender, n (%)*	
Female	10 (83.3)
Male	2 (16.7)

*AS* ankylosing spondylitis, *CD* Crohn’s disease, *PsA* psoriatic arthritis, *RA* rheumatoid arthritis, *SD* standard deviation

### Psychometric assessment of the ASI

#### Psychometric analysis sample

Of the 70 subjects who were screened for participation in the RA0098 clinical study, 36 had RA, 15 had CD, 11 had PsA, 7 had ankylosing spondylitis (AS), and 1 disease report was missing. Mean age was 52 years (SD: 13 years) and approximately half of patients were in the Q2W schedule treatment group (53%; 37/70). At Visit 1, 68 (100%) patients completed both the pre- and post-injection ASI modules; at Visit 2, 68 (100%) patients completed the pre-injection module, of whom 65 (95.6%) completed the post-injection module; 3 patients did not complete the post-injection module [16].

#### Construct validity

CFA was performed on all ASI items, using the original SIAQ item groupings (Additional file 6: Table S6) [12]. In the pre-injection module, items were well correlated with their hypothesized domain; all standardized loadings were > 0.6 and modification indices (MIs) did not show any potential meaningful improvement to the model. Some items in the post-injection module did not correlate well with their hypothesized domains: items included in the Pain and Skin Reactions domain did not fit well together; “how difficult or easy was it to travel with the PFS/PFP/e-Device?” did not fit well with other items in the domain; “how easy was it to give yourself an injection with the PFS/PFP/e-Device?” had a better fit with the Ease of Use domain than with the Satisfaction domain.

Based on the CFA results, several changes were made to the grouping of items in the post-injection ASI domains. Items included in the Pain and Skin Reactions domain were separated as single items, rather than aggregated in a domain score. The item “how difficult or easy was it to travel with the PFS/PFP/e-Device?” was removed from the Ease of Use domain as it was not considered relevant during the qualitative interviews. One item from the Satisfaction domain, “how easy was it to give yourself an injection with the (PFS/PFP/e-Device)?” had a better fit with the Ease of Use domain and was therefore included in this domain score. Following the changes, all fit indices (except RMR) improved; standardized loadings were > 0.6 for all items and MIs did not show any conceptually relevant improvements to the model.

RMT analyses were conducted on all items included in both the pre- and post-injection ASI components and at both visits. Across all items and domains, gaps were observed along the continuum and floor effects were recorded. No disordered thresholds were observed for items included in any pre-injection domains; however, in the post-injection module, disordered thresholds were observed for two of the three Self-Image domain items, all items included in the Pain and Skin Reactions domain, and 15/17 items in the Ease of Use domain. For the pre-injection module, the Feelings about Injections and Self-Confidence domains had modest reliability, with PSIs of 0.69 and 0.68–0.81, respectively. For the post-injection module, the Feelings about Injections and Self-Confidence domains showed modest and good PSIs of 0.72–0.74 and 0.87–0.88, respectively. The post-injection Self-Image and Pain and Skin Reactions domains showed modest and poor PSIs of 0.50 and 0.22, respectively. The Ease of Use domain showed good reliability with a PSI of 0.82–0.88, and the Satisfaction domain had a modest PSI of 0.61–0.74.

#### Assessment of the ASI psychometric properties

Except for the Ease of Use, Satisfaction, Feelings About Injection and Self-Image domains, all other domains of both modules showed floor or ceiling effects at Visit 1 (Table 3). Visit 2 scores were similarly distributed (data not shown). Internal consistency reliability was good for all pre- and post-injection modules of the ASI (ɑ > 0.8; Table 3). Convergent and discriminant validity of the ASI was also satisfactory, with all items having moderate to high correlations with their own domain (Spearman correlation coefficients ranging from 0.58 to 0.97), and higher correlations with their own domain than with other domains (Additional file 7: Table S7). The only exception was item “How easy was it to give yourself an injection with the PFS/PFP/e-Device?” which had a higher correlation with the Satisfaction domain (r = 0.75) than with the Ease of Use domain (r = 0.63).

**Table 3:** Cross-sectional results of the ASI domains

Domain	N	n (% at floor)	n (% at ceiling)	Cronbach’s alpha	ICC	Standardized effect-size
*Pre-injection module*^a^						
Feelings about injection	68	18 (26.5)	0 (0.0)	0.83	–	–
Self-confidence	68	9 (13.2)	12 (17.6)	0.95	–	–
*Post-injection module*						
Feelings about injection	67	23 (34.3)	0 (0.0)	0.88	0.78	− 0.03
Self-image	67	31 (46.3)	3 (4.5)	0.88	0.44	− 0.05
Self-confidence	67	12 (17.9)	25 (37.3)	0.98	0.50	0.29
Ease of use	67	0 (0.0)	19 (28.4)	0.96	0.59	0.09
Satisfaction	66	0 (0.0)	29 (43.9)	0.93	0.69	0.11

*ASI* assessment of self-injection, *ICC* intraclass correlation coefficient

^a^The Satisfaction domain of the pre-injection ASI module was not included in the pilot ASI administered in the RA0098 clinical study, thus it was not possible to assess the psychometric properties of this domain. Scores for each domain were between 0 (lowest possible i.e. floor) and 10 (highest possible i.e. ceiling)

Known-groups validity for pre- and post-injection ASI scores at Visit 1 and Visit 2 was assessed for each age group, gender, disease and dosing schedule group. There were no statistically significant differences in the pre-injection domain scores, however, at Visit 1, the post-injection Self-Confidence domain scores were significantly higher for males than for females (p = 0.028). There were also differences in mean scores across diseases in the Ease of Use domain (p = 0.010; 9.52 for AS, 9.70 for CD, 9.17 for PsA and 8.70 for RA). Also, for this domain, there was a significantly higher mean score in the Q4W group (9.23; SD 1.48) than in the Q2W group (8.96; SD 1.05; p = 0.047). Similarly, the Satisfaction domain score was significantly higher in the Q4W group than in the Q2W group (p = 0.030). At Visit 2, the only significant difference observed was in the Ease of Use domain, with a higher mean score in the Q4W group (9.42; SD 0.92) than in the Q2W group (9.00; SD 0.95; p = 0.026).

Test–retest reliability was unsatisfactory for most post-injection ASI scores (Table 3). The ICC for the Feelings About Injection domain score was 0.78. The ICC of the other post-injection ASI scores were < 0.70, ranging from 0.44 for the Self-Image score to 0.69 for the Satisfaction score. ES results were negligible (< 0.2) to small (0.2 to < 0.5) for all scores; of note, ES and SRM were small but consistent between Q2W and Q4W groups for the Self-Confidence domain scores.

## Discussion

Developing PRO instruments that appropriately and accurately capture patient experience requires thoughtful review of the concepts of interest and the specific context of use. The selection and adaptation of the SIAQ was based on substantial research; the SIAQ was developed in a similar context (i.e. for patients with RA who self-inject their treatment) and covered many of the important concepts identified from the literature and sponsor research. Additionally, the SIAQ contained pre- and post-injection modules which provided a suitable basis for adaptation to include aspects of self-injecting using new devices with enhanced features. The initial conceptual framework and pilot PRO instrument were revised based on qualitative patient-centered research to ensure inclusion of all relevant concepts.

Data collected in the RA0098 clinical study provided an opportunity to evaluate the properties of the ASI instrument. Exit interviews demonstrated that the ASI was acceptable and well understood by patients and was conceptually relevant to their experience of using the e-Device. The results of the preliminary psychometric analyses suggest that the ASI instrument had good cross-sectional psychometric properties. Results from the CFA analyses indicated that there were no major issues with the ASI content structure and items accurately reflected their underlying concepts. RMT analysis revealed disordered thresholds suggesting that the response scale for some items may not be appropriate. This could be addressed in future use of the ASI by considering certain items in isolation.

Longitudinal analyses did not demonstrate strong test–retest reliability. However, given the study design of RA0098, these results were likely; although patients’ health status can be considered the same at each visit, the conditions in which the ASI was assessed were not the same. At Visit 1, patients completed the post-injection ASI after self-injecting using the e-Device for the first time; whereas at Visit 2, patients completed the ASI having self-injected using the e-Device twice or four times, depending on their dosing schedule. Consequently, their expectations, for example fear of injecting using the e-Device, may have shifted between the two visits, explaining why the intraclass correlation coefficients did not reach the recommended threshold. Metrics showed low responsiveness and were consistent across dosing schedule groups; this could be due to mostly positive scores at Visit 1, which left little margin for improvement at Visit 2. In the future, it would be interesting to assess the psychometric properties of the ASI in another context with a study design allowing more substantial changes over time or greater differences between two or more groups to be detected.

Strengths of this study include the multiple different methods and steps (qualitative literature review, focus groups, exit interviews, quantitative psychometric analysis) of the ASI instrument’s iterative development process, which facilitated incorporation of several different perspectives. Consultation with patients facilitated inclusion of patient-relevant concepts. Further, the ASI instrument was developed through adaptation of a previously used and validated PRO instrument; the SIAQ has been previously demonstrated to be a valid and reliable tool for assessing patient experience of self-injection [17]. Cross-sectional psychometric findings in this study were broadly similar to those published in the SIAQ validation study, indicating that the ASI bears good potential for use in clinical contexts [17].

Potential limitations of this research include the small sample size of qualitative data obtained in the focus groups. Further, patients who participated in the focus groups were recruited from a single geographic location, and so may not have been representative of wider populations of patients who self-inject their treatment. Additionally, some items included in the ASI assess aspects of self-injection that were only hypothetical in the population interviewed. For example, patients could not experience limitations associated with traveling with the e-Device because the devices remained on-site at the clinic. Finally, the design of the RA0098 clinical study did not facilitate longitudinal data collection, given that the study included just two site visits at close timepoints.

## Conclusions

Key concepts relevant to patients who subcutaneously self-inject treatment were compared with those included in existing PRO instruments. Based on a targeted literature review, the SIAQ provided a suitable basis for adaptation to fully assess patient experience of self-injecting using new devices with advanced features [17]. The ASI was then developed iteratively with patient input from focus groups and exit interviews, and administered in a clinical context, as is the recommended best practice for PRO development [13, 14]. The instrument was considered relevant and well understood by patients during the exit interviews. These findings suggest the ASI instrument is appropriate for use in real‑world settings to aid with clinical decision making.

### Supplementary information

**Additional file 1: Table S1.** Literature review search terms: self-injection concepts and PRO instruments. *PRO* patient-reported outcome.

**Additional file 2: Table S2.** Comparison of the advantages and disadvantages of an e-Device vs a PFS. *PFS* pre-filled syringe.

**Additional file 3: Table S3.** Final version of the ASI. *ASI* Assessment of Self-Injection.

**Additional file 4: Table S4.** Adaptation of the SIAQ and development of the ASI. *ASI* Assessment of Self-Injection, *PFS* pre-filled syringe, *SIAQ* Self-Injection Assessment Questionnaire.

**Additional file 5: Table S5.** Exit interview schedule.

**Additional file 6: Table S6.** Fit summary of CFA on the pre- and post-injection ASI modules. *AGFI* adjusted goodness-of-fit index, *ASI* Assessment of Self-Injection, *CFA* confirmatory factor analysis, *GFI* goodness-of-fit index, *RMR* root mean square residual, *RMSEA* root mean square error of approximation, *SRMR* standardized root mean square.

**Additional file 7: Table S7.** Discriminant validity of the ASI (Spearman correlation coefficients between ASI items and scores). In bold, correlations of the item with its own domain. *ASI* Assessment of Self-Injection

## Data Availability

Underlying data from this manuscript may be requested by qualified researchers six months after product approval in the US and/or Europe, or global development is discontinued, and 18 months after trial completion. Investigators may request access to anonymized IPD and redacted study documents which may include: raw datasets, analysis-ready datasets, study protocol, blank case report form, annotated case report form, statistical analysis plan, dataset specifications, and clinical study report. Prior to use of the data, proposals need to be approved by an independent review panel at www.Vivli.org and a signed data sharing agreement will need to be executed. All documents are available in English only, for a pre-specified time, typically 12 months, on a password protected portal.

## Appendix: Assessment of self-injection copyright and use

The ASI is copyrighted by UCB Biopharma SRL and, should you be interested in using the ASI, the questionnaire use is free of charge but requires explicit written permission to use from UCB Biopharma SRL for each study. This permission will be granted if the study sponsor agrees to the conditions mentioned below (an agreement by email is sufficient).

The following conditions apply for the permission to use the ASI:

1. The permission to use from UCB Biopharma SRL must be acknowledged in any publication as follows: "The ASI questionnaire is used by [Name of Sponsor] with the permission of the copyright holder, UCB Biopharma SRL, S.A., Belgium".
2. UCB Biopharma SRL should be given the opportunity to comment, from a PRO methodology perspective, on any abstracts, papers and other peer-reviewed communications resulting from the use of the ASI, prior to their submission.
3. Any additional linguistic adaptations must be developed, at a minimum, according to the methodological standards defined in Acquadro et al. (2004) Mapi Research Institute (Ed.) [27]. As developer of the questionnaire, UCB Biopharma SRL must be consulted from a methodological standpoint in these adaptations.
4. A royalty-free, non-exclusive, perpetual license to use all newly-produced translations in any indication must be granted to UCB Biopharma SRL and its affiliates worldwide.

## Abbreviations

AGFI: Adjusted goodness of fit index
AS: Ankylosing spondylitis
Anti-TNF: Anti-tumor necrosis factor
ASI: Assessment of self injection
axSpA: Axial spondyloarthritis
CZP: Certolizumab pegol
CFA: Confirmatory factor analysis
CD: Crohn’s disease
Q2W: Every two weeks
Q4W: Every four weeks
GFI: Goodness of fit index
IPAQ: Injection Pen Assessment Questionnaire
ES: Effect size
NFI: Normed fit index
PFP: Pre-filled pen
PFS: Pre-filled syringe
PRO: Patient reported outcomes
PSO: Psoriasis
PsA: Psoriatic arthritis
RMT: Rasch measurement theory
RA: Rheumatoid arthritis
RMSEA: Root mean square error of approximation
RMR: Root mean square residual
SIAQ: Self-Injection Assessment Questionnaire
SRM: Standardized response mean
TRIM: Treatment-related impact measures
