# Supplementary material for: Development and psychometric evaluation of the assessment of self-injection questionnaire: an adaptation of the self-injection assessment questionnaire
Source: Health Qual Life Outcomes. 2020 Nov 4;18:355. doi: 10.1186/s12955-020-01606-7 (PMC7640481; doi:10.1186/s12955-020-01606-7)
Supplement: Supplementary file 1 — Additional file 1: Table S1. Literature review search terms: self-injection concepts and PRO instruments. PRO patient-reported outcome. [file 12955_2020_1606_MOESM1_ESM.docx]

**SUPPLEMENTARY MATERIAL**

**Supplementary Table S1.** Literature review search terms: self-injection concepts and PRO instruments

|  | **Search Terms** | |
| --- | --- | --- |
|  | **Clinical Study** | **Observational Study** |
| **Concepts** | (((device) OR (self-inject*) OR (autoinject*) OR (auto-inject*) OR (e-inject*) OR (self-inject*) OR (electronic inject*) OR (electromechanical inject*) OR (automated inject*) OR (electromechanical device) OR (electronic device) OR (autoinjection) OR (self-injection devices))) AND (((patient experience) OR (patient satisfaction) OR (patient benefit) OR (patient preference))) AND (((qualitative) OR (conceptual framework) OR (interviews))) AND ("last 10 years"] AND Humans) | (((device) OR (self-inject*) OR (autoinject*) OR (auto-inject*) OR (e-inject*) OR (self-inject*) OR (electronic inject*) OR (electromechanical inject*) OR (automated inject*) OR (electromechanical device) OR (electronic device) OR (autoinjection) OR (self-injection devices))) AND (((adherence) OR (compliance) OR (persistency) OR (drug survival) OR (discontinuation rate) OR (treatment continuation))) AND (((qualitative) OR (conceptual framework) OR (interviews))) AND ("last 10 years" AND Humans) |
| **PRO Instruments** | (((device) OR (self-inject*) OR (autoinject*) OR (auto-inject*) OR (e-inject*) OR (self-inject*) OR (electronic inject*) OR (electromechanical inject*) OR (automated inject*) OR (electromechanical device) OR (electronic device) OR (autoinjection) OR (self-injection devices))) AND (((patient experience) OR (patient satisfaction) OR (patient benefit))) AND (((instrument) OR (scale) OR (patient reported outcome measure) AND (patient reported experience measure))) AND ("last 10 years" AND Humans) | (((device) OR (self-inject*) OR (autoinject*) OR (auto-inject*) OR (e-inject*) OR (self-inject*) OR (electronic inject*) OR (electromechanical inject*) OR (automated inject*) OR (electromechanical device) OR (electronic device) OR (autoinjection) OR (self-injection devices))) AND (((adherence) OR (compliance) OR (persistency) OR (drug survival) OR (discontinuation rate) OR (treatment continuation))) AND (((instrument) OR (scale) OR (patient reported outcome measure) AND (patient reported experience measure))) AND ("last 10 years" AND Humans) |

PRO: patient-reported outcome.
