# Supplementary material for: Development and psychometric evaluation of the assessment of self-injection questionnaire: an adaptation of the self-injection assessment questionnaire
Source: Health Qual Life Outcomes. 2020 Nov 4;18:355. doi: 10.1186/s12955-020-01606-7 (PMC7640481; doi:10.1186/s12955-020-01606-7)
Supplement: Supplementary file 2 — Additional file 2: Table S2. Comparison of the advantages and disadvantages of an e-Device vs a PFS. PFS pre-filled syringe. [file 12955_2020_1606_MOESM2_ESM.docx]

Supplementary Table S2. Comparison of the advantages and disadvantages of an e-Device vs a PFS

|  | e-Device | | | PFS | |
| --- | --- | --- | --- | --- | --- |
|  | **Pros** | **Cons** | **Pros** | | **Cons** |
| Ease of use | - Removing needle cup appears very easy - Simpler disposal of needle when in cassette - Self-injecting with one hand (vs need for both hands) - No need to pinch the skin - Self-injecting using thumb - Easier to get correct angle/wrong angle more pain - Accuracy of speed - Accessories “more transportable” | - More cumbersome - More complicated to transport/more stuff to carry when traveling - Overall longer process - Would only work for home use, not when traveling - When self-injecting in public, people will wonder what you are doing | - Overall ease with  self-injection using design - Quicker/simpler | | - Difficulty in removing needle cover (due to grip or older age) - Difficulty self-injecting at required angle - Difficult/awkward to manipulate (this was expressed by both naïve and experienced patients) - More difficult to use as you can only use one hand - Need to fold/pinch the skin |
| Safety | - Disposal of needle when in cassette |  |  | | - Exposed needles (both during storage and disposal) around patients’ children |
| Features | - Memory and calendar reminders - Enjoy gadgets in general - Needle masked - Provision of instructions - Adjustable speed – can affect pain levels | - Bulkier/heavier - not handy for travel - Beeping noise/sound that cannot be turned down or switched off - Reliability of operating system (electronic machines can fail) - Reliability of memory/calendar (electronic vs hard copies) - More costly |  | | - Volume of exposed medicine can be daunting - PFS described as “violent” |
| Reaction | - Potentially less pain/more comfortable due to needle masking/touch of skin - Potentially less itching | - Potentially more pain due to no pinching |  | | - Angle/speed influencing pain experienced, which is variable |

PFS: pre-filled syringe.
