# Supplementary material for: Development and psychometric evaluation of the assessment of self-injection questionnaire: an adaptation of the self-injection assessment questionnaire
Source: Health Qual Life Outcomes. 2020 Nov 4;18:355. doi: 10.1186/s12955-020-01606-7 (PMC7640481; doi:10.1186/s12955-020-01606-7)
Supplement: Supplementary file 3 — Additional file 3: Table S3. Final version of the ASI. ASI Assessment of Self-Injection. [file 12955_2020_1606_MOESM3_ESM.docx]

Supplementary S3. Final version of the ASI

*Pre-injection module*

**Feelings about injections**

1. In general, how afraid are you of needles?

| Not at all  1 | A little  2 | Moderately  3 | Very  4 | Extremely  5 |
| --- | --- | --- | --- | --- |

1. In general, how afraid are you of having an injection?

| Not at all  1 | A little  2 | Moderately  3 | Very  4 | Extremely  5 |
| --- | --- | --- | --- | --- |

1. How anxious do you feel about giving **yourself** an injection?

| Not at all  1 | A little  2 | Moderately  3 | Very  4 | Extremely  5 |
| --- | --- | --- | --- | --- |

**Self-confidence**

1. How confident are you about:

|  | **Not at all** | **A little** | **Moderately** | **Very** | **Extremely** |
| --- | --- | --- | --- | --- | --- |
| 1. Giving yourself an injection in **the right way**? | 1 | 2 | 3 | 4 | 5 |
| 1. Giving yourself an injection in a **clean and sterile way**? | 1 | 2 | 3 | 4 | 5 |
| 1. Giving yourself an injection **safely**? | 1 | 2 | 3 | 4 | 5 |

**Satisfaction**

1. Overall how satisfied are you with your current method of self-injecting your medication?

| Very dissatisfied  1 | Dissatisfied  2 | Neither satisfied nor dissatisfied  3 | Satisfied  4 | Very satisfied  5 |
| --- | --- | --- | --- | --- |

*Post-injection module*

**Feelings about injections**

1. In general, how afraid are you of needles?

| Not at all  1 | A little  2 | Moderately  3 | Very  3 | Extremely  4 |
| --- | --- | --- | --- | --- |

1. In general, how afraid are you of having an injection?

| Not at all  1 | A little  2 | Moderately  3 | Very  4 | Extremely  5 |
| --- | --- | --- | --- | --- |

1. How anxious do you feel about giving yourself an injection?

| Not at all  1 | A little  2 | Moderately  3 | Very  4 | Extremely  5 |
| --- | --- | --- | --- | --- |

**Self-image**

1. How **self-conscious** would you feel about using the prefilled syringe/AutoClicks prefilled pen/ava^®^:

|  | **Not at all** | **A little** | **Moderately** | **Very** | **Extremely** |
| --- | --- | --- | --- | --- | --- |
| 1. … around your **family**? | 1 | 2 | 3 | 4 | 5 |
| 1. … around your **friends**? | 1 | 2 | 3 | 4 | 5 |
| 1. … around **people you don’t know**? | 1 | 2 | 3 | 4 | 5 |

**Self-confidence**

1. How **confident** are you about:

|  | **Not at all** | **A little** | **Moderately** | **Very** | **Extremely** |
| --- | --- | --- | --- | --- | --- |
| 1. Giving yourself an injection in **the right way**? | 1 | 2 | 3 | 4 | 5 |
| 1. Giving yourself an injection in a **clean and sterile way**? | 1 | 2 | 3 | 4 | 5 |
| 1. Giving yourself an injection **safely**? | 1 | 2 | 3 | 4 | 5 |

**Pain and skin reactions during or after the injection**

1. During and/or after the injection, how **bothered** were you by:

|  | **Not at all** | **A little** | **Moderately** | **Very** | **Extremely** |
| --- | --- | --- | --- | --- | --- |
| 1. **pain**? | 1 | 2 | 3 | 4 | 5 |
| 1. **burning sensation**? | 1 | 2 | 3 | 4 | 5 |
| 1. **cold sensation**? | 1 | 2 | 3 | 4 | 5 |
| 1. **itching** at the injection site? | 1 | 2 | 3 | 4 | 5 |
| 1. **redness** at the injection site? | 1 | 2 | 3 | 4 | 5 |
| 1. **swelling** at the injection site? | 1 | 2 | 3 | 4 | 5 |
| 1. **bruising** at the injection site? | 1 | 2 | 3 | 4 | 5 |
| 1. **hardening** at the injection site? | 1 | 2 | 3 | 4 | 5 |
| 1. **bleeding** from the injection site? | 1 | 2 | 3 | 4 | 5 |
| 1. **medication leaking from the skin** at the injection site? | 1 | 2 | 3 | 4 | 5 |

**Ease of Use of the self-injection device**

1. How **difficult** or **easy** was it to:

|  | **Very difficult** | **Difficult** | **Somewhat difficult** | **Somewhat easy** | **Easy** | **Very easy** |
| --- | --- | --- | --- | --- | --- | --- |
| 1. read and follow the prefilled syringe/ AutoClicks prefilled pen/ava^®^ **instructions**? | 1 | 2 | 3 | 4 | 5 | 6 |
| 1. learn **how to use** the prefilled syringe/AutoClicks prefilled pen/ava^®^? | 1 | 2 | 3 | 4 | 5 | 6 |
| 1. Remove the **needle cap** of the prefilled syringe/AutoClicks prefilled pen/ava^®^? | 1 | 2 | 3 | 4 | 5 | 6 |
| 1. **hold** the prefilled syringe/AutoClicks prefilled pen/ava^®^ while preparing it and giving yourself medication? | 1 | 2 | 3 | 4 | 5 | 6 |
| 1. hold the prefilled syringe/AutoClicks prefilled pen/ava^®^ at the **correct angle** for injection? | 1 | 2 | 3 | 4 | 5 | 6 |
| 1. depress the **plunger or button** on the prefilled syringe/ AutoClicks prefilled pen/ava^®^? | 1 | 2 | 3 | 4 | 5 | 6 |
| 1. **administer** the injection without any help? | 1 | 2 | 3 | 4 | 5 | 6 |
| 1. control the injection **speed**? | 1 | 2 | 3 | 4 | 5 | 6 |
| 1. **pause** when giving yourself an injection? | 1 | 2 | 3 | 4 | 5 | 6 |
| 1. **stop** when giving yourself an injection? | 1 | 2 | 3 | 4 | 5 | 6 |
| 1. be sure that the injection gave you the **correct amount** of medication? | 1 | 2 | 3 | 4 | 5 | 6 |
| 1. know when the injection is **complete**? | 1 | 2 | 3 | 4 | 5 | 6 |
| 1. **remember** when to take my **next** injection? | 1 | 2 | 3 | 4 | 5 | 6 |
| 1. **store** the prefilled syringe AutoClicks prefilled pen/ava^®^? | 1 | 2 | 3 | 4 | 5 | 6 |
| 1. **travel** with the prefilled syringe/ AutoClicks prefilled pen/ava^®^ | 1 | 2 | 3 | 4 | 5 | 6 |
| 1. **use** the prefilled syringe/AutoClicks prefilled pen/ava^®^? | 1 | 2 | 3 | 4 | 5 | 6 |

1. How does the [prefilled syringe/AutoClicks prefilled pen/ava^®^] **fit in your hand**?

| Very uncomfortably  1 | Uncomfortably  2 | Somewhat uncomfortably  3 | Somewhat comfortably  4 | Comfortably  5 | Very comfortably  6 |
| --- | --- | --- | --- | --- | --- |

**Satisfaction with self-injection**

1. How **satisfied** are you with the way the [prefilled syringe/AutoClicks prefilled pen/ava^®^] **delivers** your **medication** (syringe needle or medication cassette)?

| Very dissatisfied  1 | Dissatisfied  2 | Neither dissatisfied nor satisfied  3 | Satisfied  4 | Very satisfied  5 |
| --- | --- | --- | --- | --- |

1. After this study, how **confident** would you be to give yourself injections at home with the [prefilled syringe/AutoClicks prefilled pen/ava^®^]?

| Not at all  1 | A little  2 | Moderately  3 | Very  4 | Extremely  5 |
| --- | --- | --- | --- | --- |

1. How **easy** was it to give yourself an injection with the [prefilled syringe/AutoClicks prefilled pen/ava^®^]?

| Not at all  1 | A little  2 | Moderately  3 | Very  4 | Extremely  5 |
| --- | --- | --- | --- | --- |

1. How satisfied are you with your **ability to control** your injection (e.g. stop, pause, change speed) with the [prefilled syringe/AutoClicks prefilled pen/ava^®^]?

| Very dissatisfied  1 | Dissatisfied  2 | Neither dissatisfied nor satisfied  3 | Satisfied  4 | Very satisfied  5 |
| --- | --- | --- | --- | --- |

1. How satisfied are you with the **time it takes to inject the medication with the** [prefilled syringe/AutoClicks prefilled pen/ava^®^]?

| Very dissatisfied  1 | Dissatisfied  2 | Neither dissatisfied nor satisfied  3 | Satisfied  4 | Very satisfied  5 |
| --- | --- | --- | --- | --- |

1. Overall, how **convenient** is the [prefilled syringe/AutoClicks prefilled pen/ava^®^]?

| Very inconvenient  1 | Inconvenient  2 | Neither inconvenient nor convenient  3 | Convenient  4 | Very convenient  5 |
| --- | --- | --- | --- | --- |

1. After this study, would you **choose to continue** self-injecting your medication with the [prefilled syringe/AutoClicks prefilled pen/ava^®^]?

| Definitely not  1 | Probably not  2 | I don’t know  3 | Yes, probably  4 | Yes, definitely  5 |
| --- | --- | --- | --- | --- |

1. Overall, how **satisfied** are you with the [Prefilled syringe/AutoClicks prefilled pen/ava^®^]?

| Very dissatisfied  1 | Dissatisfied  2 | Neither dissatisfied nor satisfied  3 | Satisfied  4 | Very satisfied  5 |
| --- | --- | --- | --- | --- |
