# Supplementary material for: Development and psychometric evaluation of the assessment of self-injection questionnaire: an adaptation of the self-injection assessment questionnaire
Source: Health Qual Life Outcomes. 2020 Nov 4;18:355. doi: 10.1186/s12955-020-01606-7 (PMC7640481; doi:10.1186/s12955-020-01606-7)
Supplement: Supplementary file 4 — Additional file 4: Table S4. Adaptation of the SIAQ and development of the ASI. ASI Assessment of Self-Injection, PFS pre-filled syringe, SIAQ Self-Injection Assessment Questionnaire. [file 12955_2020_1606_MOESM4_ESM.docx]

**Supplementary Table S4**. Adaptation of the SIAQ and development of the ASI

| **Stage:** | **ASI Module:** | **Change made:** |
| --- | --- | --- |
| **Pre-Focus Group 1** | Pre-injection | - One item was deleted (“How satisfied are you with your current way of taking your medication?”) |
|  | Post-injection | - One item was deleted from the self-confidence domain (“Are you confident you could stop or pause injection?”) - Two items were added to burden from injection site reactions domain (“bleeding at injection site”; “medication leaking from injection site”) - One item was deleted (“prepare the device for use”) from the ease of use domain - Three items were added (“remove needle cap”; “hold at correct angle for injection”; “travel with device”) to the ease of use domain - Also, in the ease of use domain, the “pause/stop” item was split into two separate questions - Some items on the satisfaction domain were reworded to clarify ambiguity reported by patients: items about being “embarrassed” or “uncomfortable” were changed to be “How self-conscious would you feel about using the device?”) - Formatting changes to the instrument were completed for improved readability and clarity, with a clearer font, number responses rather than tick-boxes, use of page breaks, item groups and shading to separate like items |
| **Post-Focus Group 1, pre-Focus Group 2** | Pre- and post-injection | - Headers and shading added - Addition of “bleeding at injection site” - Replacement of “prepare device for use” with “remove needle cap” and “hold at correct angle for injection” - “Pause” and “stop” separated as individual items - Addition of “travel with the device” - Replacement of “control the way you get your medication” with “control your injection” - Alteration of “the way PFS/e-Device gives you your medication” to the way PFS/e-Device delivers your medication (syringe needle or medication cassette)” |
| **Post-Focus Group 2, pre-RA0098** | Pre- and post-injection | - Alteration of “how embarrassed would you feel if someone saw you with the [PFS/e-Device]?” to “how self-conscious would you feel if someone saw you with the [PFS/e-Device] a) around family, b) around your friends, c) around people who you don’t know?”, thus merging the following item “how uncomfortable would you feel about using the [PFS/e-Device] in front of other people?” |

ASI: Assessment of Self-Injection; PFS: pre-filled syringe; SIAQ: Self-Injection Assessment Questionnaire.
