# Supplementary material for: Development and psychometric evaluation of the assessment of self-injection questionnaire: an adaptation of the self-injection assessment questionnaire
Source: Health Qual Life Outcomes. 2020 Nov 4;18:355. doi: 10.1186/s12955-020-01606-7 (PMC7640481; doi:10.1186/s12955-020-01606-7)
Supplement: Supplementary file 5 — Additional file 5: Table S5. Exit interview schedule. [file 12955_2020_1606_MOESM5_ESM.docx]

**Supplementary Table S5.** Exit interview schedule

| **Patient ID** | **Exit interview date (MM/DD/YYYY)** |
| --- | --- |
| PATIENT-01 | 1/3/2018 |
| PATIENT-02 | 2/9/2018 |
| PATIENT-03 | 1/25/2018 |
| PATIENT-04 | 2/1/2018 |
| PATIENT-05 | 2/15/2018 |
| PATIENT-06 | 2/22/2018 |
| PATIENT-07 | 2/22/2018 |
| PATIENT-08 | 3/1/2018 |
| PATIENT-09 | 3/7/2018 |
| PATIENT-10 | 3/20/2018 |
| PATIENT-11 | 3/15/2018 |
| PATIENT-12 | 4/13/2018 |
