# Supplementary material for: Development and psychometric evaluation of the assessment of self-injection questionnaire: an adaptation of the self-injection assessment questionnaire
Source: Health Qual Life Outcomes. 2020 Nov 4;18:355. doi: 10.1186/s12955-020-01606-7 (PMC7640481; doi:10.1186/s12955-020-01606-7)
Supplement: Supplementary file 6 — Additional file 6: Table S6. Fit summary of CFA on the pre- and post-injection ASI modules. AGFI adjusted goodness-of-fit index, ASI Assessment of Self-Injection, CFA confirmatory factor analysis, GFI goodness-of-fit index, RMR root mean square residual, RMSEA root mean square error of approximation, SRMR standardized root mean square. [file 12955_2020_1606_MOESM6_ESM.docx]

**Supplementary Table S6.** Fit summary of CFA on the pre- and post-injection ASI modules

| **Fit index** | **Fit** | | |
| --- | --- | --- | --- |
| **Pre-injection module** | | | |
| RMR | 0.0889 | | |
| SRMR | 0.0570 | | |
| GFI | 0.9543 | | |
| AGFI | 0.8799 | | |
| RMSEA | 0.0612 | | |
| **Post-injection module** | **Model 1** | **Model 2** | **Model 3** |
| RMR | 0.0862 | 0.0764 | 0.0813 |
| SRMR | 0.1139 | 0.1056 | 0.0899 |
| GFI | 0.4801 | 0.4892 | 0.5357 |
| AGFI | 0.4197 | 0.4282 | 0.4630 |
| RMSEA | 0.1544 | 0.1459 | 0.1447 |

AGFI: adjusted goodness-of-fit index; ASI: Assessment of Self-Injection; CFA: confirmatory factor analysis; GFI: goodness-of-fit index; RMR: root mean square residual; RMSEA: root mean square error of approximation; SRMR: standardized root mean square.
