# Supplementary material for: Development and psychometric evaluation of the assessment of self-injection questionnaire: an adaptation of the self-injection assessment questionnaire
Source: Health Qual Life Outcomes. 2020 Nov 4;18:355. doi: 10.1186/s12955-020-01606-7 (PMC7640481; doi:10.1186/s12955-020-01606-7)
Supplement: Supplementary file 7 — Additional file 7: Table S7. Discriminant validity of the ASI (Spearman correlation coefficients between ASI items and scores). In bold, correlations of the item with its own domain. ASI Assessment of Self-Injection [file 12955_2020_1606_MOESM7_ESM.docx]

**Supplementary Table S7.** Discriminant validity of the ASI (Spearman correlation coefficients between ASI items and scores)

| **Domains** | **Items** | **Pre-injection: feelings about injections score** | **Pre-injection: self-confidence score** | **Post-injection: feelings about injection score** | **Post-injection: self-image score** | **Post-injection: self-confidence score** | **Post-injection: ease of use of the self-injection device score** | **Post-injection: satisfaction with self-injection score** |
| --- | --- | --- | --- | --- | --- | --- | --- | --- |
| Pre-injection: feelings about injections | In general, how afraid are you of needles? | **0.87** | -0.01 | 0.72 | 0.33 | -0.10 | -0.27 | -0.36 |
|  | In general, how afraid are you of having an injection? | **0.83** | -0.06 | 0.70 | 0.23 | -0.10 | -0.25 | -0.31 |
|  | How anxious do you feel about giving yourself an injection? | **0.83** | -0.21 | 0.73 | 0.18 | -0.33 | -0.39 | -0.27 |
| Pre-injection: self-confidence | Giving yourself an injection in the right way? | -0.14 | **0.94** | -0.01 | 0.08 | 0.62 | 0.33 | 0.10 |
|  | Giving yourself an injection in a clean and sterile way? | -0.02 | **0.93** | 0.06 | 0.12 | 0.56 | 0.25 | 0.08 |
|  | Giving yourself an injection safely? | -0.13 | **0.97** | -0.04 | 0.02 | 0.64 | 0.35 | 0.13 |
| Post-injection: feelings about injections | In general, how afraid are you of needles? | 0.78 | 0.07 | **0.88** | 0.45 | -0.06 | -0.31 | -0.33 |
|  | In general, how afraid are you of having an injection? | 0.75 | 0.06 | **0.86** | 0.23 | -0.14 | -0.34 | -0.31 |
|  | How anxious do you feel about giving yourself an injection? | 0.74 | -0.09 | **0.89** | 0.24 | -0.30 | -0.51 | -0.46 |
| Post-injection: self-image | How self-conscious would you feel about using the prefilled syringe / AutoClicks prefilled pen / ava® around your family? | 0.17 | 0.11 | 0.21 | **0.67** | 0.14 | -0.06 | 0.02 |
|  | How self-conscious would you feel about using the prefilled syringe / AutoClicks prefilled pen / ava® around your friends? | 0.20 | 0.10 | 0.25 | **0.85** | 0.13 | -0.10 | -0.13 |
|  | How self-conscious would you feel about using the prefilled syringe / AutoClicks prefilled pen / ava® around people you don't know? | 0.30 | 0.06 | 0.34 | **0.97** | 0.19 | -0.11 | -0.22 |
| Post-injection: self-confidence | Giving yourself an injection in the right way? | -0.21 | 0.58 | -0.20 | 0.17 | **0.96** | 0.47 | 0.30 |
|  | Giving yourself an injection in a clean and sterile way? | -0.12 | 0.65 | -0.07 | 0.21 | **0.94** | 0.40 | 0.23 |
|  | Giving yourself an injection safely? | -0.16 | 0.63 | -0.17 | 0.20 | **0.97** | 0.45 | 0.26 |
| Post-injection: ease of use of the self-injection device | read and follow the prefilled syringe/AutoClicks prefilled pen/ava® instructions? | -0.28 | 0.42 | -0.32 | -0.05 | 0.49 | **0.82** | 0.38 |
|  | learn how to use the prefilled syringe/AutoClicks prefilled pen/ava®? | -0.28 | 0.26 | -0.35 | -0.04 | 0.38 | **0.77** | 0.41 |
|  | Remove the needle cap of the prefilled syringe/AutoClicks prefilled pen/ava®? | -0.28 | 0.30 | -0.31 | -0.07 | 0.30 | **0.78** | 0.37 |
|  | hold the prefilled syringe/AutoClicks prefilled pen/ava® while preparing it and giving yourself medication? | -0.20 | 0.29 | -0.30 | -0.04 | 0.46 | **0.79** | 0.33 |
|  | hold the prefilled syringe/AutoClicks prefilled pen/ava® at the correct angle for injection? | -0.17 | 0.28 | -0.26 | -0.02 | 0.41 | **0.79** | 0.31 |
|  | depress the plunger or button on the prefilled syringe/AutoClicks prefilled pen/ava®? | -0.31 | 0.20 | -0.41 | -0.03 | 0.44 | **0.77** | 0.39 |
|  | administer the injection without any help? | -0.40 | 0.29 | -0.44 | -0.09 | 0.42 | **0.76** | 0.50 |
|  | control the injection speed? | -0.19 | 0.24 | -0.23 | -0.14 | 0.24 | **0.73** | 0.47 |
|  | pause when giving yourself an injection? | -0.40 | 0.30 | -0.49 | -0.19 | 0.40 | **0.78** | 0.46 |
|  | stop when giving yourself an injection? | -0.32 | 0.35 | -0.34 | -0.06 | 0.48 | **0.82** | 0.46 |
|  | be sure that the injection gave you the correct amount of medication? | -0.36 | 0.14 | -0.43 | -0.06 | 0.35 | **0.70** | 0.37 |
|  | know when the injection is complete? | -0.34 | 0.16 | -0.40 | -0.12 | 0.31 | **0.65** | 0.39 |
|  | remember when to take my next injection? | -0.33 | 0.14 | -0.39 | -0.10 | 0.34 | **0.69** | 0.36 |
|  | store the prefilled syringe/AutoClicks prefilled pen/ava®? | -0.34 | 0.32 | -0.33 | -0.08 | 0.41 | **0.74** | 0.43 |
|  | use the prefilled syringe/AutoClicks prefilled pen/ava®? | -0.19 | 0.34 | -0.23 | -0.02 | 0.41 | **0.75** | 0.51 |
|  | How does the prefilled syringe/AutoClicks prefilled pen/ava® fit in your hand? | -0.21 | 0.01 | -0.30 | -0.07 | 0.20 | **0.58** | 0.46 |
|  | How easy was it to give yourself an injection with the prefilled syringe/AutoClicks prefilled pen/ava®? | -0.39 | 0.21 | -0.41 | -0.21 | 0.32 | **0.63** | 0.75 |
| Post-injection: satisfaction with self-injection | How satisfied are you with the way the prefilled syringe/AutoClicks prefilled pen/ava® delivers your medication (syringe needle or medication cassette)? | -0.27 | 0.11 | -0.32 | -0.15 | 0.26 | 0.44 | **0.80** |
|  | After this study, how confident would you be to give yourself injections at home with the prefilled syringe/AutoClicks prefilled pen/ava®? | -0.36 | 0.28 | -0.33 | -0.08 | 0.40 | 0.65 | **0.70** |
|  | How satisfied are you with your ability to control your injection (eg, stop, pause, change speed) with the prefilled syringe/AutoClicks prefilled pen/ava®? | -0.28 | 0.18 | -0.33 | -0.09 | 0.36 | 0.61 | **0.89** |
|  | How satisfied are you with the time it takes to inject the medication with the prefilled syringe/AutoClicks prefilled pen/ava®? | -0.27 | 0.20 | -0.32 | -0.11 | 0.31 | 0.54 | **0.84** |
|  | Overall, how convenient is the prefilled syringe/AutoClicks prefilled pen/ava®? | -0.26 | 0.12 | -0.25 | -0.12 | 0.17 | 0.44 | **0.69** |
|  | After this study, would you choose to continue self-injecting your medication with the prefilled syringe/AutoClicks prefilled pen/ava®? | -0.21 | -0.01 | -0.24 | -0.26 | 0.16 | 0.36 | **0.79** |
|  | Overall, how satisfied are you with the prefilled syringe/AutoClicks prefilled pen/ava®? | -0.25 | -0.00 | -0.29 | -0.07 | 0.12 | 0.37 | **0.70** |

In bold, correlations of the item with its own domain. ASI: Assessment of Self-Injection.
